# Integrating Gemcitabine-Based Therapy With AdipoRon Enhances Growth Inhibition in Human PDAC Cell Lines

**DOI:** 10.3389/fphar.2022.837503

**Published:** 2022-02-22

**Authors:** Angela Ragone, Alessia Salzillo, Annamaria Spina, Silvio Naviglio, Luigi Sapio

**Affiliations:** Department of Precision Medicine, University of Campania “Luigi Vanvitelli”, Naples, Italy

**Keywords:** PDAC, AdipoRon, gemcitabine, cell cycle, P44/42 MAPK, drug resistance

## Abstract

Pancreatic ductal adenocarcinoma (PDAC) accounts for 90% of all pancreatic cancers. Albeit its incidence does not score among the highest in cancer, PDAC prognosis is tremendously fatal. As a result of either aggressiveness or metastatic stage at diagnosis, chemotherapy constitutes the only marginally effective therapeutic approach. As gemcitabine (Gem) is still the cornerstone for PDAC management, the low response rate and the onset of resistant mechanisms claim for additional therapeutic strategies. The first synthetic orally active adiponectin receptor agonist AdipoRon (AdipoR) has recently been proposed as an anticancer agent in several tumors, including PDAC. To further address the AdipoR therapeutic potential, herein we investigated its pharmacodynamic interaction with Gem in human PDAC cell lines. Surprisingly, their simultaneous administration revealed a more effective action in contrasting PDAC cell growth and limiting clonogenic potential than single ones. Moreover, the combination AdipoR plus Gem persisted in being effective even in Gem-resistant MIA PaCa-2 cells. While a different ability in braking cell cycle progression between AdipoR and Gem supported their cooperating features in PDAC, mechanistically, PD98059-mediated p44/42 MAPK ablation hindered combination effectiveness. Taken together, our findings propose AdipoR as a suitable partner in Gem-based therapy and recognize the p44/42 MAPK pathway as potentially involved in combination outcomes.

## Introduction

According to Global Cancer Statistics 2020, pancreatic cancer (PC) ranks the seventh leading cause of cancer death worldwide, with an estimated 466,003 deaths against 495,773 new cases (Collisson et al., 2019; Sung et al., 2021). Although its incidence rate and the number of casualties do not reach the top score of cancers, PC is currently considered one of the most aggressive malignancies due to a rapidly progressive and fatal prognosis (Carioli et al., 2021).

Arising from either ductal or acinar cells of the exocrine portion, pancreatic ductal adenocarcinoma (PDAC) accounts for 90% of all pancreatic cancers, while the remainder chiefly evolves from Langerhans islets (Gao et al., 2020). While this latter subtype is typically linked to an abnormal hormone secretion even at the early stage, facilitating its detection and diagnosis, PDAC is almost a symptom-free disease until metastases, or rather when the advanced stage leaves no longer chances of recovery (Mpilla et al., 2020).

In addition to the histological characterization, molecular subtyping is essentially guiding preclinical and clinical therapeutic strategies and treatment in malignancies, including leukemia and breast and colorectal cancers (Esposito et al., 2015; Verret et al., 2020; Singh et al., 2021; Tewari et al., 2021). Collecting the existing molecular data, a similar subgroup grading has recently been made even in PDAC (Collisson et al., 2019). Albeit quite promising, this therapeutic approach has not been fully translated in clinical yet; thus, chemotherapy still remains the best option for curing PDAC patients (Qian et al., 2020). Indeed, considering the advanced and metastasized stages at diagnosis, the surgical resection rate remains very low in PDAC (Huang et al., 2019).

Two distinct chemotherapeutic regimens currently recognize the first-line approach in progressive PDAC, namely, FOLFIRINOX (folinic acid, 5-fluorouracil, irinotecan, oxaliplatin) and gemcitabine (Gem) plus nab-paclitaxel (Riedl et al., 2021). Although FOLFIRINOX provides significant results in improving both overall and median progression-free survival, its toxicity drastically restricts administration for patients with good performance status (Damm et al., 2021). Therefore, either alone or in combination, Gem remains the standard of care for advanced PDAC, as well as neoadjuvant therapy (Oba et al., 2020). Regrettably, the limited toxicity and the extensive usage of Gem usually conflict with a very low response rate and resistant mechanism acquisition (Amrutkar and Gladhaug, 2017; Fu et al., 2021).

Despite the huge efforts made to improve prevention and treatment over the years, only weak signs of progress have been obtained in PDAC, where prognosis still remains extremely poor with a less than 10% 5-year survival rate (Collisson et al., 2019). Moreover, recent perspective reports indicate a harsh increase in both incidence and mortality rates in the next two decades, making PDAC the primary cause of cancer-related death in the near future (Christenson et al., 2020). Therefore, identifying novel therapeutic approaches is absolutely mandatory in an attempt to counteract the PDAC ascent.

An increasing number of studies have provided consistent evidence supporting the potential anticancer role of AdipoRon (AdipoR) in several preclinical cancer models, including myeloma and breast, prostate, and ovarian cancers (Nigro et al., 2021). More recently, we also described how AdipoR can energetically inhibit cell proliferation in osteosarcoma cells (Sapio et al., 2020). As a synthetic orally active adiponectin receptor agonist, AdipoR exerts comparable pharmacological properties to those of its template, such as anti-obesity, antidiabetic, and anti-ischemic features (Nigro et al., 2021). Antineoplastic effects have been reported even in PDAC where, delaying cell cycle progression in the G0/G1 phase, AdipoR induces both *in vitro* and *in vivo* growth arrest (Messaggio et al., 2017; Akimoto et al., 2018). The assessment of the AdipoR-mediated mechanisms has revealed the involvement of AMPK dependent and independent pathways in PDAC. Precisely, beyond the canonical activation of AMPK and its related downstream target acetyl-CoA carboxylase (ACC), AdipoR has been described to module pathways as signal transducer and activator of transcription 3 (STAT3), protein kinase B (PKB), extracellular signal-regulated kinase 1/2 (ERK1/2), and p38 (Messaggio et al., 2017; Akimoto et al., 2018).

Taking the outlined state of art into account, the present study has been conceived to further explore the AdipoR relevance in PDAC therapy. Specifically, since no data currently provide information on the AdipoR plus Gem combination outcome, herein we addressed potential cooperating effects between these two compounds in PDAC. Using MIA PaCa-2 and PANC-1 as human PDAC cell lines, combinatory and single drug effectiveness was evaluated by multiple methodological approaches. Starting from the biological results, estimated by cell growth and colony forming assays, we characterized the cell phase distribution and initially investigated the molecular mechanisms underlying single and combination stimulations. Finally, combination and AdipoR usefulness were further explored in MIA PaCa-2 Gem-resistant cells.

## Materials and Methods

### Cell Culture Maintenance and Drug Treatments

MIA PaCa-2 and PANC-1 human PDAC cell lines were purchased by the American Type Culture Collection (ATCC) and maintained at 37°C in a 5% CO_2_ humidified atmosphere, using Dulbecco’s Minimum Essential Medium (DMEM) (ECM0728L; Euroclone) supplemented with 10% fetal bovine serum (FBS) (ECS0180L; Euroclone) and 1% penicillin/streptomycin (ECB3001D; Euroclone) as culture medium. Typically, cells were equally seeded and kept under standard growing conditions for 24 h. The following day, AdipoR and Gem were supplemented to fresh media, either individually or in combination, and PDAC cells were incubated for times and concentrations provided in each experimental condition. Ultimately, adherent cells were trypsinized and collected with potential floating ones, before being centrifuged for 5 min at 1,500 RPM. Since AdipoRon and gemcitabine were dissolved in DMSO and H_2_O, respectively, an equal solvent rate (% v/v) was used as a negative control.

### Chemical Reagents and Antibodies

Chemicals: AdipoRon (#SML0998; Sigma-Aldrich), gemcitabine (#G6423, Sigma-Aldrich), trypan blue (#T8154; Sigma-Aldrich), propidium iodide (#P4864; Sigma-Aldrich), crystal violet (#C0775; Sigma-Aldrich), PD98059 (#P215; Sigma-Aldrich), dimethyl sulfoxide (DMSO) (A3672; AppliChem), and ethanol absolute anhydrous (308603; Carlo Erba). Antibodies: α-Tubulin (#3873; Cell Signaling Technology), cyclin E1 (#4129; Cell Signaling Technology), p44/42 MAPK (#9102; Cell Signaling Technology), phospho-p44/42 MAPK (#9101; Cell Signaling Technology), cyclin A1 (sc-751; Santa Cruz Biotechnology), vinculin (sc-73614; Santa Cruz Biotechnology), and p27^KIP1^ (ab3203; Abcam).

### Assessment of Drug-Mediated Effects on Living and Death Cells

A total number of 8 × 10^4^ MIA PaCa-2 and 1 × 10^5^ PANC-1 cells were moved in 6-well plates and kept in a standard growing state for 24 h. AdipoR and Gem, either alone or in combination, were subsequently added to new media and allowed to act in PDAC cells. For each experiment, times and concentrations are indicated in the Results section and Figure legends. Usually, pelleted cells were resuspended in 1.5 ml DMEM and diluted 1:1 with trypan blue, which, crossing damaged membrane, discriminates living from dead cells. Specifically, 10 μl of both media containing cells and blue dye (0.4%, v/v) were mixed, and the relative cell content was established using a Bürker chamber, where the number of unstained (living) and stained (dead) cells was recorded. Each point has been counted at least twice in each experimental procedure.

### Flow Cytometry Analysis

Cytometric analysis was performed to define the respective cell phase distribution in reaction to different stimuli. A procedure similar to that described in point 2.3 was applied to seed, treat, and collect PDAC cells. Subsequently, pelleted samples were resuspended first in 300 μl PBS (ECB4004lL; Euroclone) and then in 700 μl ice-cold absolute ethanol. Fixed cells were stored at −20°C until analysis. Before investigation, the samples were spun down for 5 min at 1,500 RPM and incubated with PI staining solution containing 15 μg/ml PI and 20 μg RNase A (R5503; Sigma-Aldrich) in PBS for 10 min at room temperature in the dark side. For each experimental condition, at least 20,000 events were acquired and analyzed by FACS-Celesta (BD Biosciences).

### Colony Forming Assay

PDAC cells were seeded in 6-well plates at a density of 1.5 × 10^3^ per well (MIA PaCa-2 and MIA PaCa-2 RES) or 2 × 10^3^ (PANC-1) and exposed to different times and concentrations of AdipoR, Gem, and combination (see Results for more details). At the established endpoint, media was discarded, and newly formed colonies were stained with crystal violet solution (1% aqueous solution) for 10 min. The staining solution was later removed, and wells were washed several times in distillate water. Colonies have been allowed to air dry naturally and acquired by photographic equipment. Quantification analysis has been performed by determining the optical density (OD) of dissolved colony-bound crystal violet staining in 10% acetic acid at 590 nm by an Infinite 200 PRO Microplate Reader (Tecan Life Sciences).

### Western Blotting

Depending on the target protein, an amount of 10–30 μg of total extracts was loaded and separated by sodium dodecyl sulfate–polyacrylamide gel electrophoresis (SDS-PAGE) for each sample. Subsequently, sample proteins were transferred onto nitrocellulose membranes (GEH10600008; Amersham) by the Mini Trans-Blot system (Bio-Rad Laboratories). After washing in tris-buffered saline (TBS) supplemented with 0.05% Tween 20 (TC287; HIMEDIA), films were blocked 1 h in 5% no-fat dry milk (A0530; AppliChem) aimed at covering potentially free spots into the nitrocellulose membrane. Incubation overnight at 4°C has been chosen for primary antibody binding. In the following days, horseradish peroxidase (HRP)-conjugated secondary antibodies, reacting against the related primary species, were applied to the membrane for 1 h at room temperature. Each incubation step was preceded and followed by three 5-min rinses in T-TBS. Finally, protein-related light signals were acquired by ChemiDoc™ (Bio-Rad Laboratories) using the enhanced luminol-based chemiluminescent substrate (E-IR-R301; Elabscience) as a detection system for HRP.

### Protein Extraction and Western Blotting Sample Preparation

A number of 4.8 × 10^5^ (MIA PaCa-2) or 6 × 10^5^ (PANC-1) cells were plated in 100 mm plates and left free to attach for 24 h. In the next day, media was replaced with a fresh one containing AdipoR, Gem, and the combination in doses and timelines reported in the Results section, and Figure legends. At every experimental point, cells were collected and spun down at 1,500 RPM for 5 min. Pellets were later resuspended in 3–5 volumes of RIPA buffer (R0278; Sigma-Aldrich) supplemented with protease and phosphatase inhibitors cocktail (#5872; Cell Signaling Technology). After 30 min, samples were further centrifuged at 14,000 RPM for 15 min at 4°C, and the supernatant was recovered and assessed for the relative protein content by Bradford Assay (39222; SERVA). Protein samples were first mixed 1:1 with Laemmli 2× (S3401; Sigma-Aldrich) and later boiled at 95°C for 6 min.

### Development of Gemcitabine-Resistant MIA PaCa-1 Cells

MIA PaCa-2 cells were chronically exposed to increasing Gem concentration over a period of 4 months. Specifically, starting from 1 nM, cells were cultured in media containing Gem until they grew steadily. A higher cumulative Gem dosage was subsequently applied, and the resistant procedure was repeated as long as a final concentration of 200 nM was reached. At each step, cells were amplified, harvested, and cryopreserved in liquid nitrogen or an ultralow-temperature freezer. The obtained MIA PaCa-2 Gem-resistant cells were finally cultured in drug-free medium for up to 2 weeks before performing the reported experiments.

### Statistical Analysis

Results are indicated as average value ± SD of biological independent replicates. Significance has been defined using either Student’s t-test, to compare the mean of two samples, or analysis of variance (ANOVA) followed by Turkey’s test, to discriminate differences between more than two experimental groups. In both cases, values of less than 0.05 were recognized as significant. Densitometric analyses have been carried out by ImageJ (NIH, Bethesda).

## Results

### AdipoRon Affects Cell Growth and Slows Down Cell Cycle Progression in PDAC Cells

Recently, two different studies have reported the AdipoR ability in suppressing tumor growth in PDAC (Messaggio et al., 2017; Akimoto et al., 2018). In order to extend and corroborate these findings, herein we first established the AdipoR impact in two distinct human PDAC cell lines, namely, MIA PaCa-2 and PANC-1.

**FIGURE 1:**
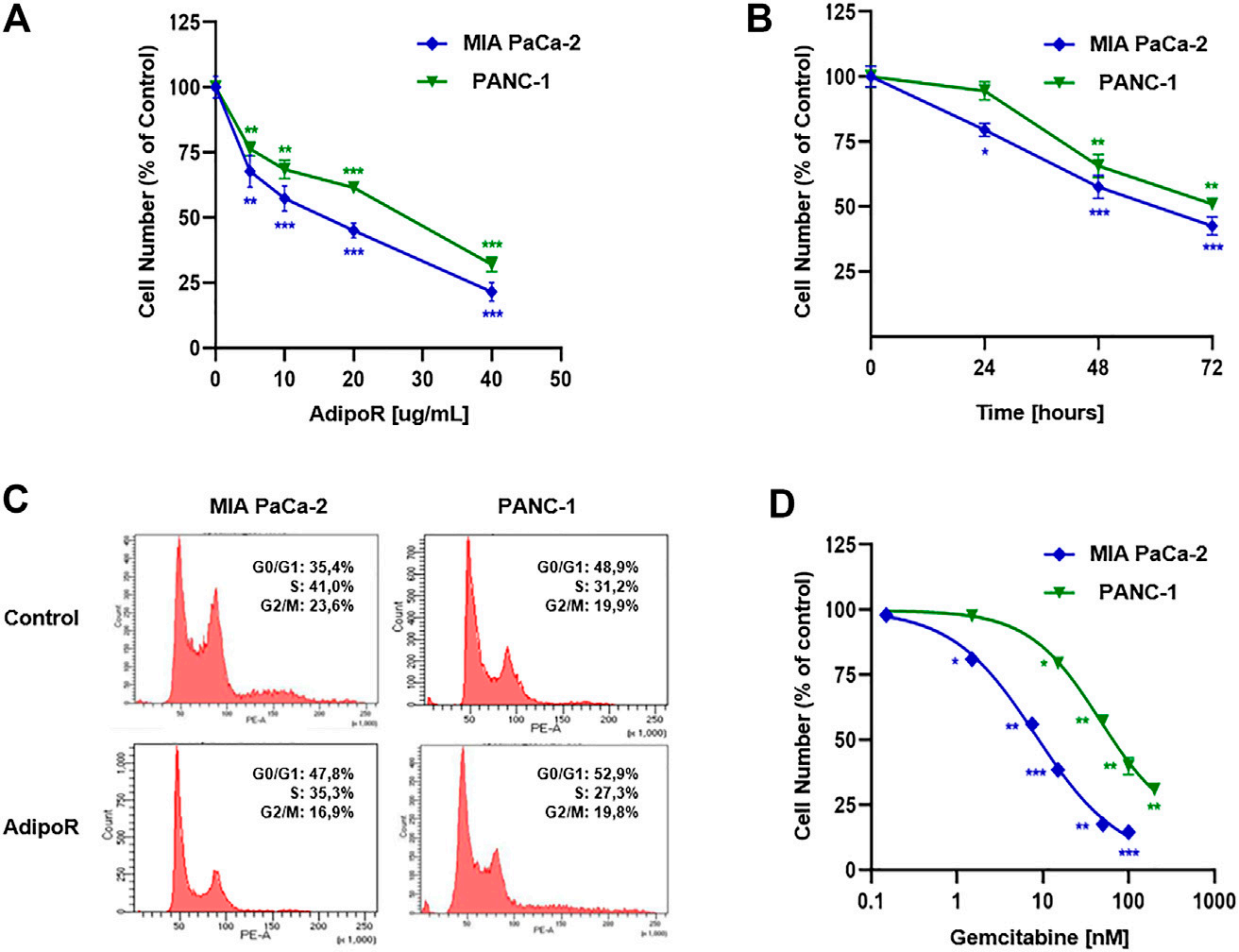
Evaluation of single drug-mediated effects in PDAC cells. **(A)** MIA PaCa-2 and PANC-1 cells were exposed for 48 h to increasing AdipoR concentrations (10–40 µg/ml). **(B)** Cell growth curves were established in reaction to 10 µg/ml AdipoR over a period of 72 h. **(C)** Representative cell cycle profiles were obtained in MIA PaCa-2 and PANC-1 cells treated and not (control) with 10 µg/ml AdipoR for 24 h. **(D)** Dose effect induced by 48 h of Gem administration in MIA PaCa-2 and PANC-1 cells. In each experimental condition, the relative cell number was estimated in triplicate and expressed in figure as % of control. *p < 0.05, **p < 0.01, ***p < 0.001 by unpaired Student’s t-test.

In agreement with the previously published results, AdipoR exposure induced a remarkable cell growth decrease in PDAC cells, almost in a dose-dependent manner, without substantial differences between MIA PaCa-2 and PANC-1 cell types (Figure 1A).

Choosing 10 µg/ml as a subsequent effective working dosage, time course experiments up to 72 h showed a near time dependency in MIA PaCa-2, where a cell number decrease of 20, 42, and 57 percent was recorded at 24, 48, and 72 h, respectively (Figure 1B). A different trend was obtained in PANC-1, in which no considerable responsiveness to AdipoR was observed at 24 h (Figure 1B).

Notably, AdipoR-mediated antiproliferative properties were supported by an increase in the G0/G1 phase and a concomitant decrease of both S and G2/M phases in MIA PaCa-2 (Figure 1C). Precisely, the cell amount in G0/G1 moved from 35 to 48% after 24 h of treatment with 10 µg/ml AdipoR, while both S and G2/M phases diminished approximately 6%, concurrently.

Interestingly, although 10 µg/ml AdipoR was not effective in impacting PANC-1 cell growth after 24 h, changes in cell cycle distribution were detected. Similar to MIA PaCa-2, AdipoR provoked a G0/G1 intensification and an S-phase depletion in PANC-1, albeit in this latter cell model the magnitude was less sharp. Conversely, no G2/M involvement seems to occur in AdipoR-treated PANC-1 cells (Figure 1C). Overall, these findings further recognize AdipoR as an antiproliferative compound in PDAC and support its peculiarity in slowing down cell cycle progression.

### Gemcitabine Influences Cell Growth With a Different Extent in PDAC Cells

Before exploring the consequences of the combination treatment AdipoR plus Gem in PDAC models, we preliminarily addressed the Gem-mediated cell growth impact on both employed cells.

Evaluating a wide concentration range, Figure 1D displays a different aptitude in reacting to Gem between MIA PaCa-2 and PANC-1. While MIA PaCa-2 showed great responsiveness to Gem already at very low concentration, the PANC-1 ability in resisting Gem was further confirmed when high dosages were applied. Exposing MIA PaCa-2 to 50 or 100 nM Gem for 48 h, for instance, nearly affected the totality of the cells, differently from PANC-1, in which the inhibition rate was roughly 40 and 60%, respectively. Taken together, these results remark an effective yet different Gem sensitivity between the examined PDAC cells.

### Combination AdipoR Plus Gem Improves Single Outcomes in PDAC Cells

With the purpose of addressing potential cooperating effects in PDAC models, we subsequently combined effective concentrations of both AdipoR and Gem in a constant dilution ratio, and the relative outcome in cell growth was later assessed.

**FIGURE 2:**
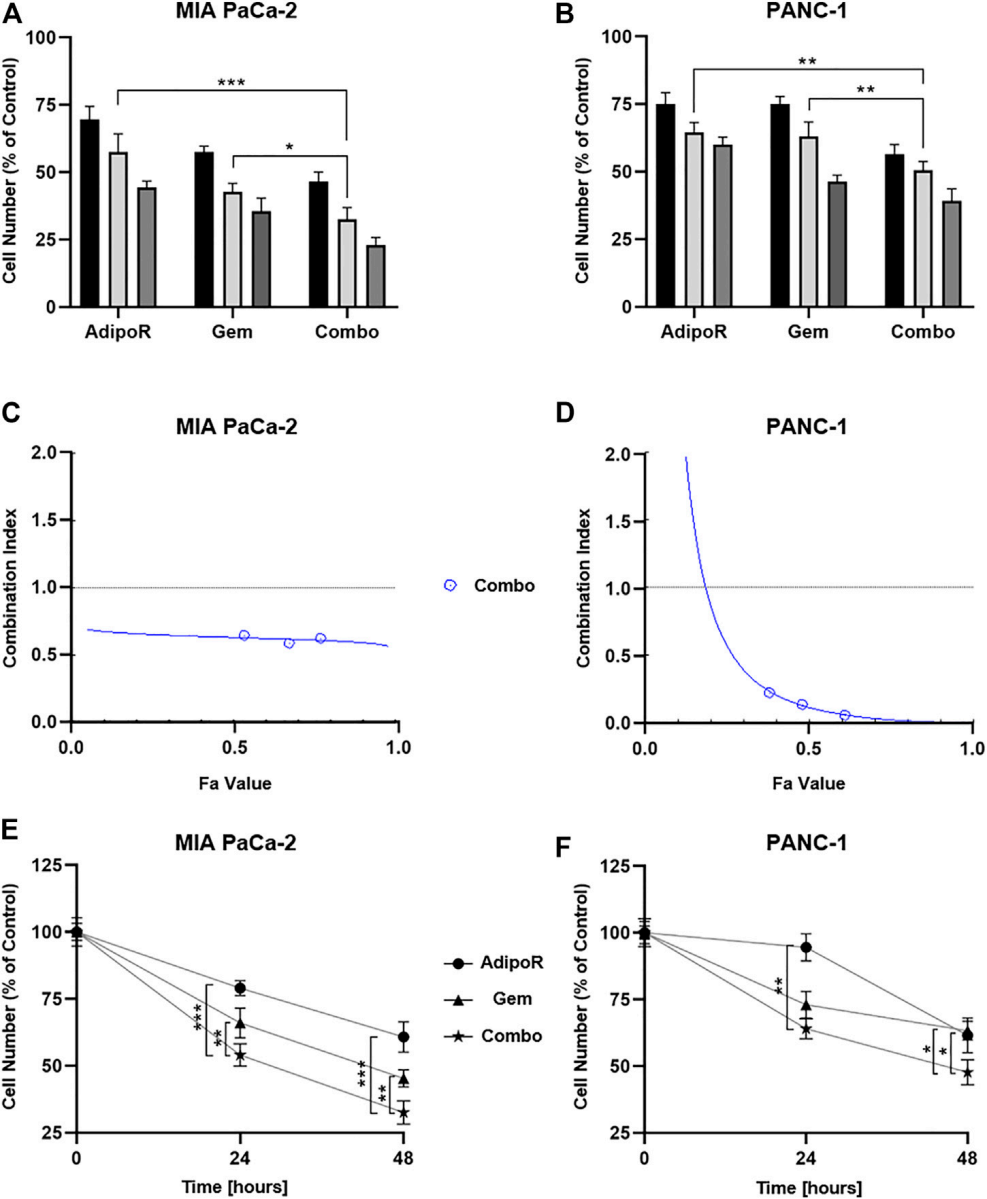
Assessment of single and combinatory outcomes in PDAC cells. **(A)** 5 (black bar), 10 (light gray), and 20 µg/ml (gray) of AdipoR were added to MIA PaCa-2 cell medium for 48 h, either alone or in combination with 7.5 (black bar), 15 (light gray), and 30 nM (gray) Gem. Colors of the columns reflect those of single drug concentrations in combination setting. **(B)** Identical AdipoR amounts were instead mixed with 25 (black bar), 50 (light gray), and 100 nM (gray) Gem in PANC-1. Representative Fa-CI report obtained in MIA PaCa-2 **(C)** and PANC-1 **(D)**. **(E)** MIA PaCa-2 growth curves achieved after 24 and 48 h under 10 µg/ml AdipoR, 15 nM Gem, and AdipoR plus Gem, respectively. The same AdipoR concentration (10 µg/ml) and a different Gem amount (50 nM) were applied in PANC-1 time course experiments **(F)**. For each stimulation, cell number was estimated at least in triplicate and reported in figure as average ± SD in % of control. *p < 0.05, **p < 0.01, ***p < 0.001 by Tukey’s multiple comparisons test.

Specifically, three different doses of both AdipoR (5, 10, and 20 µg/ml) and Gem (7.5, 15, and 30 nM) were employed in MIA PaCa-2, exhibiting a clear dose dependency (Figure 2A). But even more interestingly, the concomitant use of AdipoR plus Gem further counteracted MIA PaCa-2 cell proliferation, suggesting a positive interplay between these two compounds. Compared with 5 µg/ml AdipoR and 7.5 nM Gem, combination treatment improved single outcomes by nearly 33% and 20%, respectively. This tendency became even more pronounced at the highest tested doses, raising inhibition values of 47 and 34% versus AdipoR and Gem, individually (Figure 2A).

The different Gem responsiveness has required the use of higher concentrations in PANC-1 (25, 50, and 100 nM), while no changes in AdipoR doses were applied. In line with MIA PaCa-2 results, even in PANC-1, all three tested mixtures enhanced the anticancer effects of single treatments (Figure 2B). Minimal fluctuations were observed in response to the increasing combinations in PANC-1.

CompuSyn analysis was subsequently performed with the purpose of defining drug-drug interaction and the relative combination index (CI). Plotting dose-effect curves of both single and combination agents, the Chou-Talalay method discriminates among additive (CI = 1), synergism (CI < 1), and antagonism (CI > 1) effects, using the median-effect equation (Chou, 2010). The MIA PaCa-2 Fa-CI plot revealed a robust synergistic action already at very low concentrations, maintaining a constant trend even when combination affected 90% of cells (Figure 2C). Albeit in all tested conditions CI estimation supported a synergic action, the Fa-CI plot unveiled a different tendency in PANC-1 (Figure 2D).

Lately, we performed time-course experiments, using 10 µg/ml AdipoR plus 15 nM in MIA PaCa-2 or 50 nM Gem in PANC-1. Although co-administration AdipoR plus Gem improved single drug-mediated cell reduction in both PDAC models, different curves were outlined over time. Whilst a time dependency was revealed in reaction to both single and combination treatments in MIA PaCa-2 (Figure 2E), no clear reliance on treatment duration was observed in reaction to Gem in PANC-1. Moreover, comparing combination versus AdipoR, time exposure did not amplify the gap (Figure 2F).

Collectively, these data show that the combination of AdipoR plus Gem impairs MIA PaCa-2 and PANC-1 cell growth more effectively compared with single ones. In addition, as suggested by CompuSyn analysis, a potential synergism might exist between these two compounds.

### Co-Administration AdipoR Plus Gem Minimizes the Clonogenic Potential in PDAC Cells

The clonogenic assay is considered a valuable *in vitro* assay for monitoring undifferentiated potential and anchorage-independent growth (Rajendran and Jain, 2018). Given that Gem and AdipoR have been proved to act as effective agents in mitigating colony formation, we successively addressed the potential impact of combination AdipoR plus Gem on this PDAC feature (Messaggio et al., 2017; Alhothali et al., 2019; Zhou et al., 2019).

**FIGURE 3:**
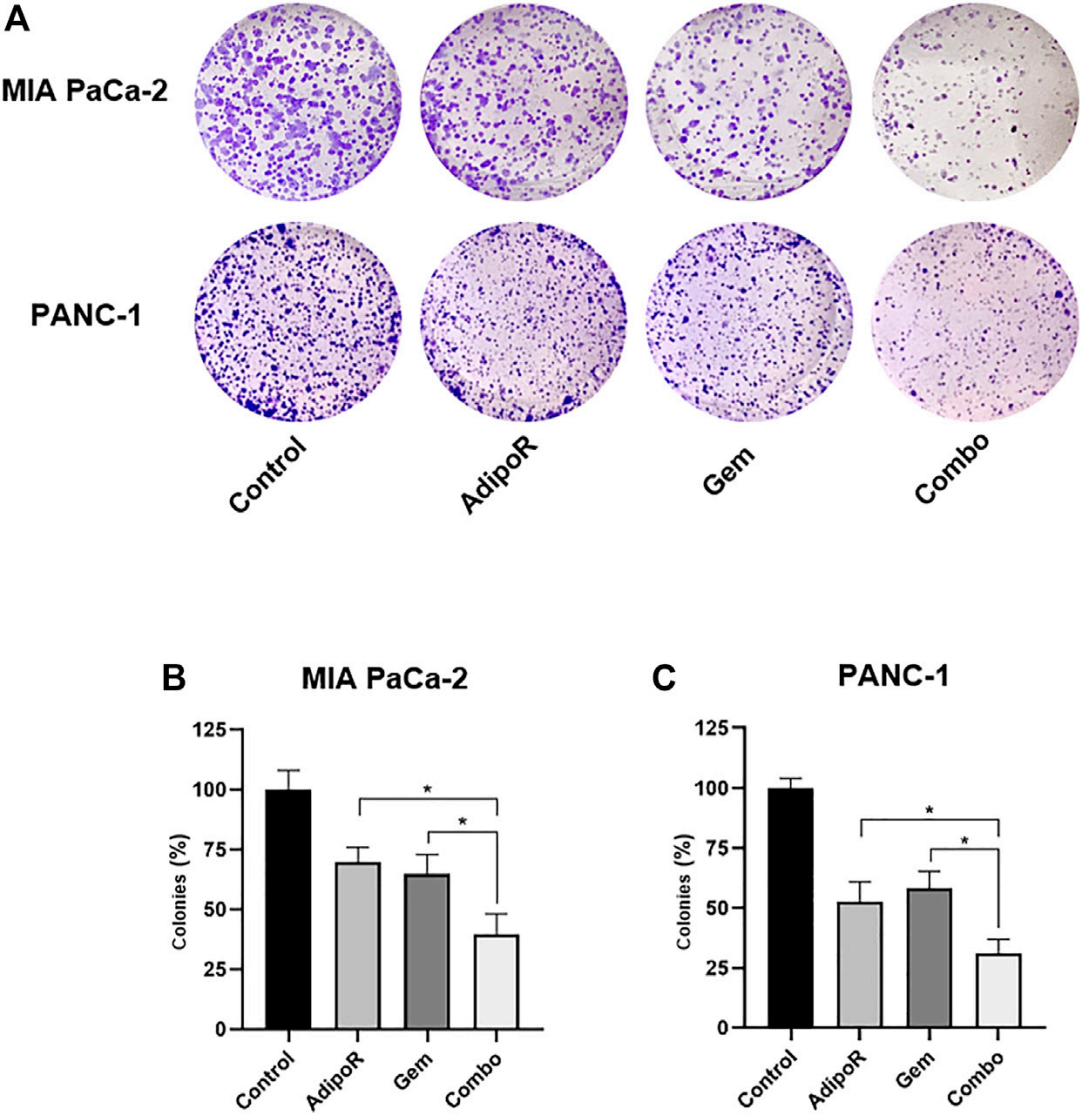
Estimation of single and combinatory impacts on clonogenic potential in PDAC cells. MIA PaCa-2 and PANC-1 were treated and not (control) with the same AdipoR concentration (2 µg/ml) but different Gem amounts (4 vs 6 nM), both individually and in combination, for 8 and 10 days, respectively. Representative stained wells are displayed in **(A)**, while the relative quantification analysis has been reported in **(B)** (MIA PaCa-2) and **(C)** (PANC-1). Experiments were reproduced thrice and plotted on a graph as mean value ± SD in % of control. *p < 0.05 by Tukey’s multiple comparisons test.

Aiming at defining the consequences of long-term exposure, PDAC cells were seeded at very low density and treated with AdipoR and Gem, both individually and in combination, until newly-formed colonies became viewable. The employment of a small amount of AdipoR and Gem moderately impaired PDAC colony-forming ability, separately (Figure 3). Conversely, a very strong reduction in PDAC clonogenic potential was observed when the same doses of AdipoR and Gem were put together (Figure 3A). Quantification analysis revealed a further enhancement in colonies reduction of nearly 40% compared to Gem alone in MIA PaCa-2, as a result of both number and size decrease (Figure 3B). Consistent results were also obtained in PANC-1 (Figure 3C).

Altogether, this evidence indicates a stronger and deeper outcome in limiting PDAC clonogenic potential made by combinatory treatment AdipoR plus Gem compared to single-agent administration.

### AdipoR Plus Gem Differently Affects Cell Cycle Phases’ Distribution in PDAC Cells

To figure out how the combination treatment AdipoR plus Gem affected PDAC cell growth, we successively performed cell cycle analysis intended to determine the cell phase distribution in reaction to our stimuli. Comprehensively, single and combination treatments were performed in both PDAC models for up to 48 h, and the relative DNA content was later detected by flow cytometry using propidium iodide (PI) as basepair intercalating dye.

**FIGURE 4:**
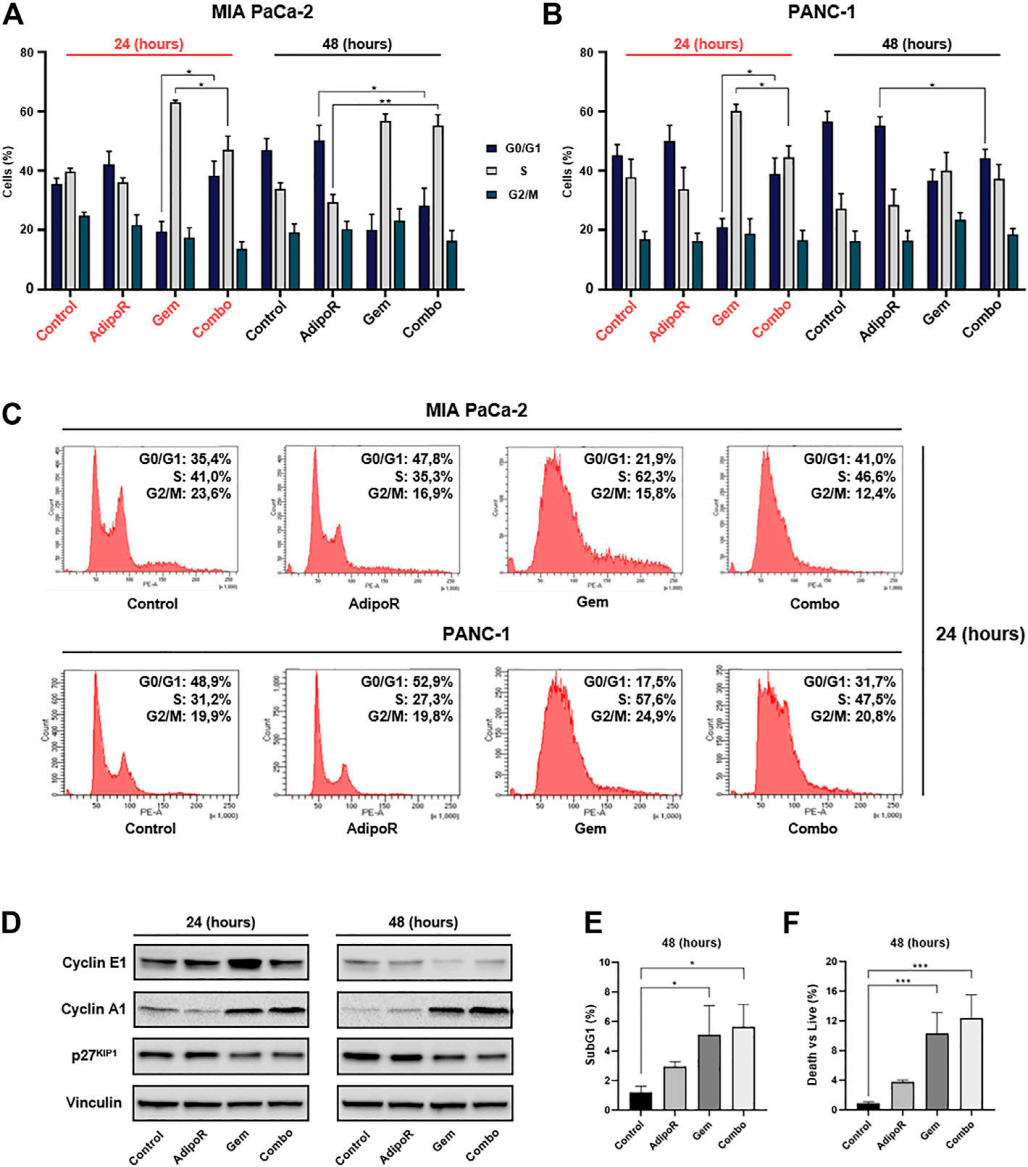
Investigation of single and combinatory consequences on cell cycle distribution in PDAC cells. MIA PaCa-2 was exposed and not (control) to 10 µg/ml AdipoR, 15 nM Gem, and AdipoR plus Gem over a period of 24 and 48 h **(A)**. PANC-1 cells, instead, were treated and not (control) with 10 µg/ml AdipoR, 50 nM Gem, and combination for the same temporal extension **(B)**. Subsequently, the relative cell phase distribution was defined by FACSCelesta^TM^ using PI as DNA staining. MIA PaCa-2 and PANC-1 representative histogram plots at 24 h **(C)**. **(D)** Cyclin A1, cyclin E1, and p27^KIP1^ expression levels were obtained in reaction to 10 µg/ml AdipoR, 15 nM Gem, and combination in MIA PaCa-2. **(E)** Relative subG1 amount. **(F)** Trypan blue discrimination analysis. Either **(E)** or **(F)** show MIA PaCa-2 results cultured in media containing AdipoR, Gem, and AdipoR plus Gem under the same **(B)** experimental conditions. Displayed data are expressed in percentage as average value ± SD of three independent experiments. *p < 0.05, **p < 0.01, ***p < 0.001 by Tukey’s multiple comparisons test.

Depending on the concentration employed, Gem has been reported to induce both S and G2 phase arrest in PDAC models (Miao et al., 2016; Montano et al., 2017; Passacantilli et al., 2018; Kumarasamy et al., 2020). In agreement with these findings, in MIA PaCa-2, we observed a remarkable S-phase accumulation in reaction to 24h Gem administration (Figures 4A,C). In respect of untreated cells, Gem raised S-phase from 40 to 62% at the expense of G0/G1 (−14%) and partly G2/M (−7%). A similar but more pronounced tendency was observed at 48 h as a result of changes in both cell density and nutrients occurring in control cells, rather than a Gem-mediated action (Figure 4A). Quite the contrary, AdipoR intensified the G0/G1 cell amount and decreased both S and G2/M phases at 24 h, while at 48 h, the G0/G1 enrichment was only supported by S-phase reduction.

Looking at the cell phase distribution in reaction to AdipoR plus Gem, different but intermediate features were detected in comparison with single agents. In this respect, after 24 h, combination displayed a G0/G1 amount closer to AdipoR, while conversely, the simultaneous presence of both AdipoR and Gem for additional 24 h exhibited an S-phase accumulation similar to Gem (Figures 4A,C). A quite comparable pattern was also obtained in PANC-1, especially following 24 h of treatment (Figures 4B,C).

In agreement with the recorded cell phase distribution, considerable changes were also detected in cyclin A1 and E1 levels, and cyclin-dependent kinase inhibitor p27^KIP1^, in reaction to both single and combined stimuli (Figure 4D; Supplementary Figure S1).

Analysis of subG1 population, which usually includes hypodiploid cells undergoing DNA fragmentation, showed a substantial increase in reaction to both Gem and combination at 48 h compared with untreated cells (Figure 4E). The absence of significant additive cytotoxic effects between Gem and combination was also confirmed in trypan blue exclusion assay, which revealed only minimal changes in death vs living cells in response to these two conditions (Figure 4F). Overall, these findings reveal a different ability in braking cell cycle progression among AdipoR, Gem, and combination.

### p44/42 MAPK Is Dynamically Involved in AdipoR Plus Gem Outcomes in PDAC Cells

As the most frequent mutated gene, abnormal KRAS hyperactivation occurs recurrently in PDAC (Buscail et al., 2020). Consequently, dysregulation of the p44/42 MAPK pathway has been recognized in PDAC, assuming a possible correlation between its expression and tumor prognosis (Furukawa, 2015).

Modulation of p44/42 MAPK has also been detected in response to Gem administration in both *in vitro* and *in vivo* PDAC models, and in patients (Jin et al., 2017; Ryu et al., 2021). Correspondingly, although the AdipoR-related molecular mechanisms remain largely unknown, its antiproliferative action has been linked to p44/42 MAPK activation in PDAC (Akimoto et al., 2018). Recently, we also observed AdipoR-mediated p44/42 MAPK stimulation in osteosarcoma cell lines (Sapio et al., 2020).

Taking into account the mentioned findings and the relevance of this pleiotropic pathway in regulating the entirety of cell functions (Guo et al., 2020), we first addressed the involvement of p44/42 MAPK in reaction to our stimuli.

With this purpose, MIA PaCa-2 and PANC-1 cells were treated with AdipoR and Gem, alone and in co-administration, for up to 48 h and subsequently analyzed for p44/42 MAPK phosphorylation status.

**FIGURE 5:**
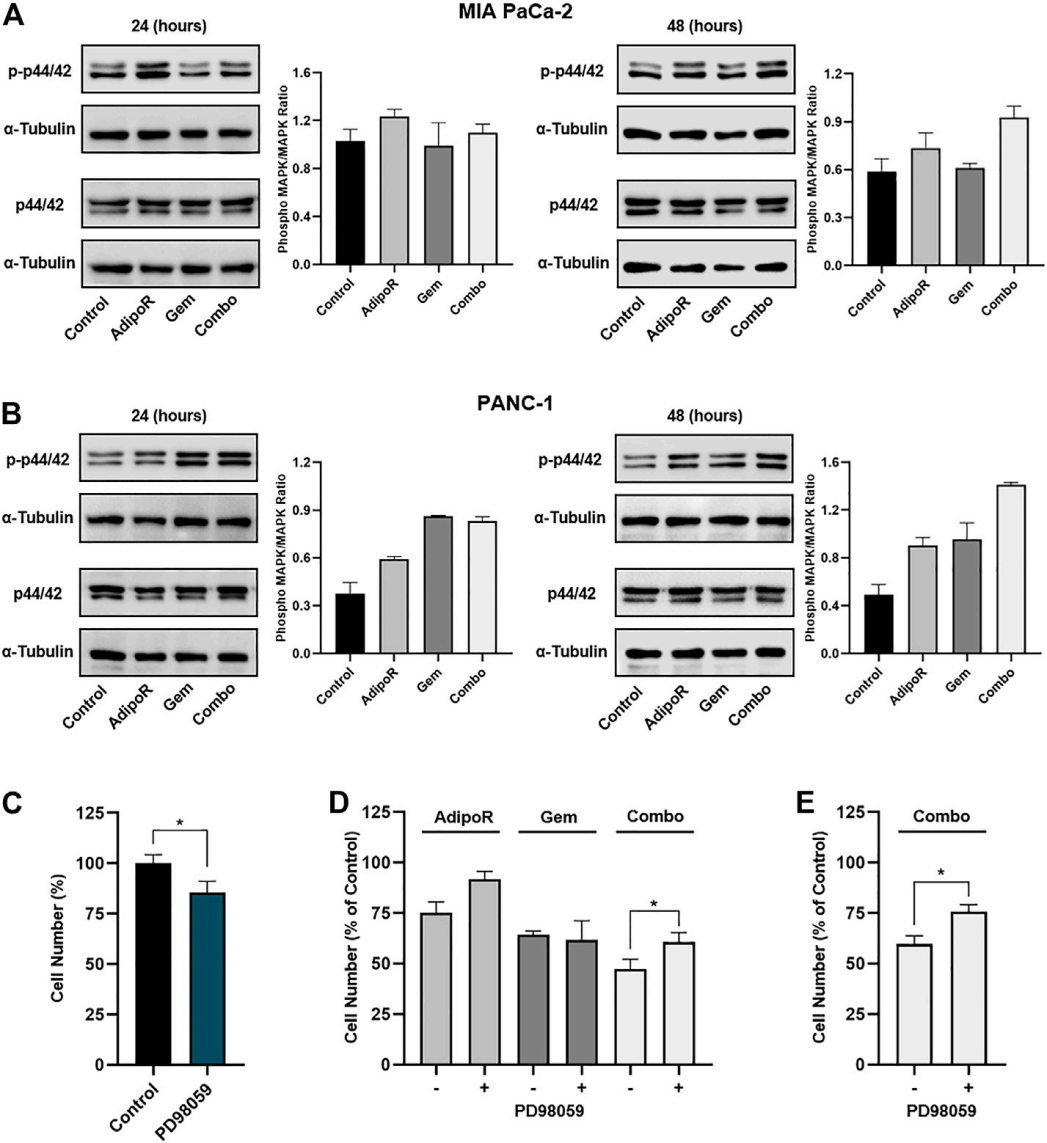
Evaluation of p44/42 MAPK involvement in AdipoR plus Gem effects. MIA PaCa-2 **(A)** and PANC-1 **(B)** were treated and not (control) with 10 µg/ml AdipoR, 15 nM (MIA PaCa-2) or 50 nM (PANC-1) Gem, and AdipoR plus Gem over a period of 48 h. Thereafter, either single or combination consequences on p44/42 MAPK activation (phosphorylation) were estimated by Western blotting. Phospho-MAPK/MAPK ratio results from the quotient of phospho-p44/42 MAPK and its relative housekeeping on the gel divided by a quotient of p44/42 and its relative α-tubulin. **(C)** MIA PaCa-2 was exposed and not (control) to 10 μM PD98059 for 24 h, and the cell growth percentage was established. **(D)** MIA PaCa-2 single and combination treatments in MAPK-proficient and -hampered background. **(E)** Combination treatments containing 10 µg/ml AdipoR plus 50 nM Gem were carried out in PANC-1 cells with or without PD98059 inhibitor. Two hours of PD98059 pretreatment preceded and not individual and combinatory administration. Results are depicted in percentage as mean ± SD of three independent experiments. *p < 0.05 by Tukey’s multiple comparisons or Student’s t-test.

In the absence of substantial protein amount variations, we recognized a different combination capability in modulating p44/42 MAPK phosphorylation between these two cell lines. Specifically, while in MIA PaCa-2, the concomitant administration of AdipoR with Gem resulted in p44/42 MAPK activation at 48 h (Figure 5A), in PANC-1, instead phospho-p44/42 MAPK upregulation was already apparent at 24 h and maintained up to 48 h (Figure 5B).

To further investigate the p44/42 MAPK involvement in combination-mediated effects, we subsequently tested the impact of MEK1/MEK2 inhibitor PD98059 on AdipoR plus Gem outcomes in MIA PaCa-2 cells. Bearing in mind that long-term exposure to downstream blockade of MAPK deeply impairs PDAC cell growth (Wong et al., 2016), we chose 10 μM for 24 h as effective dosage of PD98059 and time to mitigate p44/42 MAPK signaling and affect MIA PaCa-2 cell growth, marginally (Figure 5C; Supplementary Figure S2A).

Although the combination of AdipoR plus Gem improved cell growth inhibition compared with single ones, PD98059 partially counteracted combination effectiveness, reducing the inhibition rate of approximately 25% relative to p44/42 MAPK-proficient counterpart (Figure 5D). Comparable experiments performed in PANC-1 also revealed a PD98059-mediated capacity in hindering the combination anticancer action (Figure 5E), albeit MEK1/MEK2 inhibitor alone affected cell growth in a more effective manner with respect to MIA PaCa-2 (Supplementary Figures S2B,C).

On the whole, these findings suppose an involvement of p44/42 MAPK pathway in AdipoR plus Gem combination response.

### Combination AdipoR Plus Gem Impairs Cell Growth Even in MIA PaCa-2-Resistant Cells

Although Gem displays one of the highest response rates compared to other anticancer agents in PDAC, resistance outbreak occurs already within few weeks of initiating dosing (Amrutkar and Gladhaug, 2017). As a result of Gem-induced refractivity, PDAC generally becomes more aggressive, causing a further reduction in overall survival (Quinonero et al., 2019).

**FIGURE 6:**
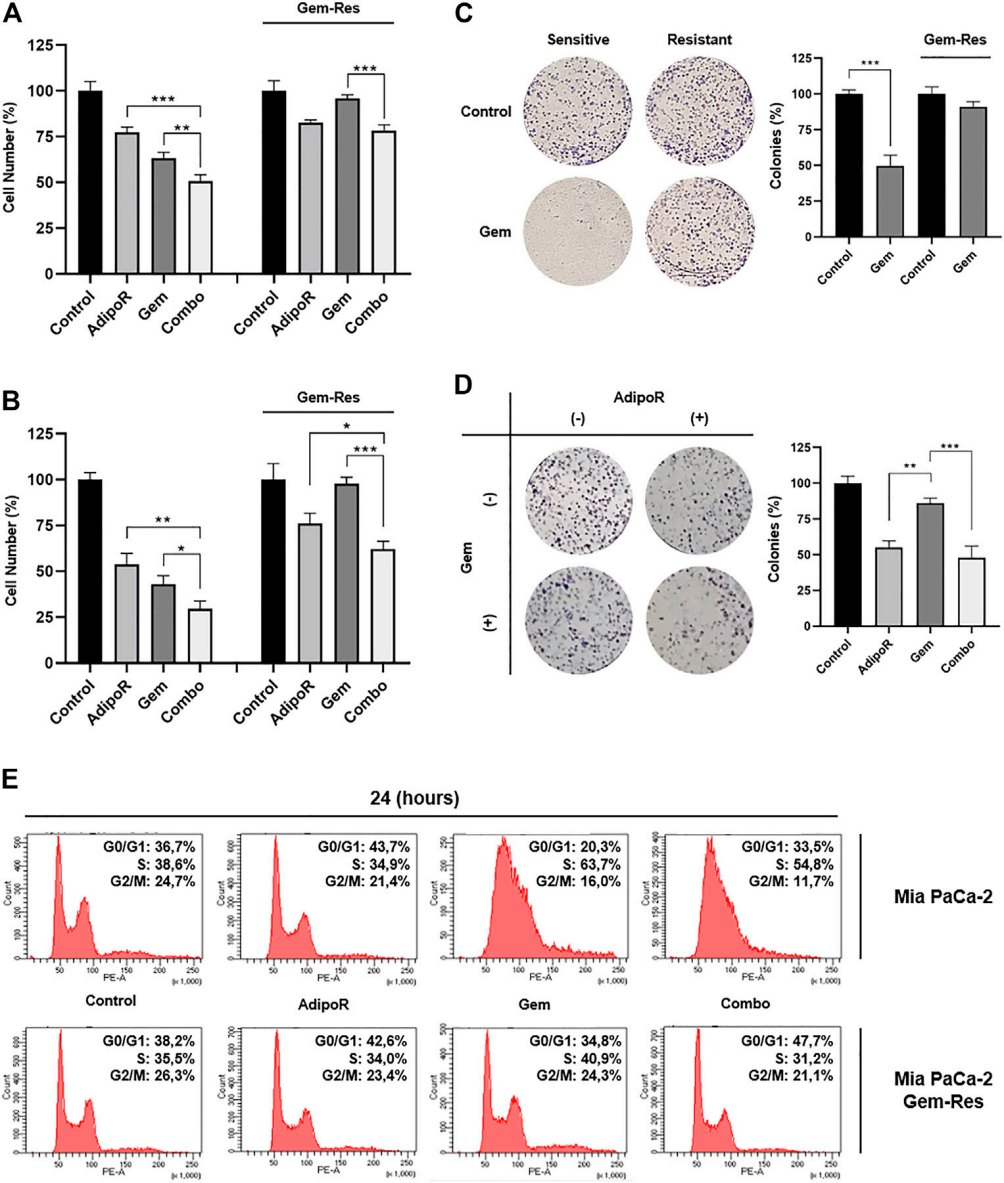
Responsiveness of MIA PaCa-2 Gem-resistant cells to single and combinatory treatments. Either MIA PaCa-2 Gem-sensitive and -resistant cells were treated and not (control) with 10 µg/ml AdipoR, 15 nM Gem, and AdipoR plus Gem for 24 **(A)** and 48 h **(B)**; thereafter, the relative impact on cell growth was addressed. **(C)** Cell media of both MIA PaCa-2 Gem-sensitive and -resistant cells were supplemented with and without (control) 10 nM Gem for 8 days. Illustrative violet-stained wells are shown on the left side, the relative quantification on the right. **(D)** MIA PaCa-2 Gem-resistant cells undergoing AdipoR (10 µg/ml) and Gem (5 nM) individually and combinatory treatments were tested for colony-forming ability. Images and quantification assay are provided in Figure. **(E)** MIA PaCa-2 Gem-sensitive and -resistant were incubated either with single or combination drugs as indicated in **(A)**. FACSCelesta^TM^ analysis was later performed with the purpose of defining the drug-induced consequences on cell phase distribution. Reported results are indicated in percentage as median value ± SD of triplicate experiments. *p < 0.05, **p < 0.01, ***p < 0.001 by Tukey’s multiple comparisons or Student’s t-test.

To further speculate the usefulness of AdipoR-based therapy in PDAC, we first developed stable MIA PaCa-2 cell lines resistant to Gem (Gem-Res). Thereafter, MIA PaCa-2 and MIA PaCa-2 Gem-Res cells were cultured in a medium containing 10 µg/ml AdipoR and 15 nM Gem, both individually and in combination for up to 48 h. As previously described in MIA PaCa-2, combination treatment resulted in a further cell growth reduction compared to AdipoR and Gem singularly, both at 24 and 48 h (Figures 6A,B). Remarkably, even though Gem was ineffective in reducing the cell number in MIA PaCa-2 Gem-Res cells, AdipoR induced a 25% growth inhibition, and even more interestingly, co-administration AdipoR plus Gem affected cell proliferation by another 18% with respect to AdipoR alone at 48 h (Figure 6B).

Using a higher dose from the one previously employed in Figure 3, Gem abrogated the colony forming ability in MIA PaCa-2, while conversely, in Gem-Res cells, the same amount marginally affected the growing colony (Figure 6C). Interestingly, either alone or in combination with Gem, AdipoR administration reduced clonogenic potential by 45 and 55%, correspondingly (Figure 6D).

Despite being less pronounced than MIA PaCa-2, flow cytometry analysis showed AdipoR persistence in braking cell cycle progression even in MIA PaCa-2 Gem-Res. Like the sensitive cells, increased G0/G1 phase was observed in the resistant ones supplemented with AdipoR (Figure 6E). But even more interesting, reducing both S and G2/M phases, the concomitant administration of AdipoR and Gem enhanced the G0/G1 accumulation compared with AdipoR alone (Figure 6E). Remarkably, no substantial changes were detected between Gem-treated and untreated cells, confirming the loss of chemotherapy responsiveness by this cell line.

Taken together, these data indicate that the combination AdipoR plus Gem is effective in preventing growth and colony formation even in Gem-resistant MIA PaCa-2 cells.

## Discussion

The existing therapeutic options have failed to provide an appropriate response in PDAC, reinforcing the unlucky privilege of being one of the deadliest cancers worldwide (Latenstein et al., 2020). Regrettably, even immunotherapy, which has recently revolutionized the drug regimes in cancer treatment (Makaremi et al., 2021), has shown few successful chances in PDAC due to tumor-related stroma abundance (Panchal et al., 2021).

Therefore, besides radiation and surgical resection, chemotherapy represents the only partially effective pharmacological approach in PDAC, irrespective of tumor stage (Qian et al., 2020). Despite the clinical approval of novel chemotherapeutics and formulations, Gem still remains a cornerstone for PDAC management, and Gem-based therapy constitutes the widely used partner in combination therapy (Christenson et al., 2020). Unfortunately, the limited success rate of Gem treatment and the relative ease in developing chemoresistance warrant for more effective therapeutic approaches in PDAC.

Recently, the first synthetic adiponectin receptor agonist is emerging as a promising anticancer compound in several tumors, including myeloma and breast, prostate, and ovarian cancers (Nigro et al., 2021). Convincing evidence is also emerging in PDAC, where AdipoR suppresses tumor growth and induces cell death, mainly through apoptosis and necroptosis induction (Messaggio et al., 2017; Akimoto et al., 2018).

With the purpose of further addressing the AdipoR candidacy in PDAC treatment, herein we investigated the potential outcome of its dynamic interaction with Gem in MIA PaCa-2 and PANC-1 cells. Albeit quite preliminary, our results reveal no shortcomings in using these two compounds together; quite to the contrary, their combination could have a greater therapeutic impact compared with single ones. Moreover, as suggested by CompuSyn analysis, potential synergistic action could exist between AdipoR and Gem. The cooperative interaction is clearly supported by cell growth and colony results, which shows a combination-mediated stronger and deeper outcome in limiting PDAC tumorigenicity. Additionally, either AdipoR or combination kept their therapeutic effectiveness even in MIA PaCa-2 cells that developed resistance to Gem administration.

Although countless other compounds have been tested over the last years, only two Gem-based combination therapies have been approved and employed in clinical for advanced PDAC treatment, namely, erlotinib and nab-Paclitaxel (Elsayed and Abdelrahim, 2021). However, while the successful rate of combination Gem plus erlotinib is strictly dependent on the EGFR status and other potential signatures (Hoyer et al., 2021), serious side effects have been reported in PDAC patients treated with Gem plus nab-paclitaxel, including neutropenia, peripheral neuropathy, and fatigue (Blomstrand et al., 2019). In addition to supporting its antineoplastic role in PDAC, our findings first recognize AdipoR as a novel potential candidate in Gem-based multidrug therapy. If subsequently confirmed by *in vivo* and trial studies, combination AdipoR plus Gem could represent an additional pharmacological choice in PDAC, especially for metastatic unresectable patients whose survival is currently under 1 year, even with an optimal chemotherapy regimen.

Mechanistically, the combination action could be explained by a different capability in slowing down cell cycle progression between AdipoR and Gem. Although in different cancer types, both Akimoto and Ramzan reported an AdipoR-mediated G0/G1 phase delay, which results in tumor growth arrest (Akimoto et al., 2018; Ramzan et al., 2019). More recently, we also observed a similar functional mechanism in the AdipoR-induced osteosarcoma stunting (Sapio et al., 2020). In agreement with the exhibiting findings, our results confirmed the ability of this compound in affecting G0/G1, as well as of Gem in blocking the S-phase (Miao et al., 2016; Montano et al., 2017; Waissi et al., 2021). Surprisingly, each compound retains its respective peculiarity even when combined. Indeed, the simultaneous administration showed intermediate features between AdipoR and Gem, wherein Gem is still arresting in S phase and AdipoR in G0/G1. Therefore, rather than inducing cytotoxic effects, our findings could suggest an experimental model in which a sum of different phase slowdown, mediated by single agents, further reduces PDAC growth.

Signaling pathway examination revealed a possible involvement of p44/42 MAPK in the responses elicited by AdipoR plus Gem in PDAC cells. In this regard, while combination stimulated p44/42 MAPK activation, PD98059-mediated p44/42 MAPK impairment partially counteracted its effectiveness. Interestingly, analog results were also observed in reaction to AdipoR, thus supposing that a proficient activation of this pathway is functional for this compound.

Different studies have reported an AdipoR-mediated p44/42 MAPK hyperphosphorylation in different pathological conditions, including in cancer (Messaggio et al., 2017; Akimoto et al., 2018). In this regard, in our previous study, we also reported how AdipoR induces a robust p44/42 MAPK activation in osteosarcoma cells (Sapio et al., 2020). In accordance with Akimoto’s results (Akimoto et al., 2018), herein we demonstrated that p44/42 MAPK activation is needed to allow a proper AdipoR antitumor action and combination outcome. Even though not in cancer models, additional studies further support the functional p44/42 MAPK role in either AdipoR- or adiponectin-mediated effects (Koskinen et al., 2011; Zhang et al., 2011; Alvarez et al., 2012; Wang et al., 2020). In this respect, Wang and coworkers have recently proved that ameliorating cell viability, apoptosis, and reactive oxygen species (ROS) production, AdipoR stimulates bone regeneration in ATDC5 cells via p44/42 MAPK pathway (Wang et al., 2020). Interestingly, when p44/42 MAPK was irreversibly suppressed by PD98059, AdipoR failed to rescue impaired apoptosis and chondrogenesis of cells. Although our results recognize this pathway as potentially involved in combination effectiveness; we cannot rule out that other signaling pathways that might be involved in, especially because the PD98059-mediated action just results in an incomplete combination rescue. In this respect, as far as known, the most common multidrug resistances are related to ATP-binding cassette (ABC) transporters, which, regulating drug absorption, distribution, and excretion, play a crucial role in overcoming drug-induced cytotoxicity (Robey et al., 2018). Recently, different ABC family members have been reported to be involved in Gem resistance, expressly in PDAC (Xu et al., 2013; Lu et al., 2019; Okada et al., 2021). Interestingly, a positive correlation between adiponectin and ABCA1 levels has been observed in visceral adipose tissue (Vincent et al., 2019). Moreover, adiponectin has been described to increase both mRNA and protein levels of ABCA1 in HepG2 hepatocellular carcinoma cells (Matsuura et al., 2007). Despite no evidence currently reports AdipoR-induced ABC modulation yet, this association could explain how this receptor agonist overcomes Gem ineffectiveness in MIA PaCa-2-resistant cells. Therefore, targeting experiments aimed at defining their relative engagement will be performed shortly.

## Conclusion

In conclusion, we first provide evidence of enhanced performances in constraining PDAC progression when AdipoR and Gem are combined. Apart from supporting the antineoplastic feature, our results recognize an additional and newly AdipoR therapeutic usage in PDAC, potentially as a partner in Gem-based combination therapy.

Considering the current orphan status for this illness, finding out novel and more effective pharmacological strategies could help in improving both PDAC prognosis and survival. In this regard, our promising *in vitro* results may encourage the development of future supplementary studies aimed at addressing the feasibility of AdipoR plus Gem approval in clinical practice.

## Data Availability

The raw data supporting the conclusion of this article will be made available by the corresponding author, without undue reservation.

## Supplementary Material

Supplementary Figure 1 Quantification analysis of the cell cycle related proteins. **(A)** Cyclin E1/Vinculin Ratio. **(B)** Cyclin A1/Vinculin Ratio. **(C)** p27^KIP1^/Vinculin Ratio. ImageJ-mediated quantification analysis has been performed processing three distinct Western blotting experiments for every cell cycle related protein, and housekeeping protein (Vinculin). Median value ± SD of the relative Ratio is reported in chart. Representative Western blotting films are displayed in Figure 4D.

Supplementary Figure 2 Effects of PD98059 inhibitor on p44/42 MAPK phosphorylation in PDAC cells. MIA PaCa-2 **(A)** and PANC-1 **(C)** were treated and not (control) with 10 μM PD98059 for 2 h with the purpose of assessing both phospho-p44/42 and p44/42 levels by Western Blotting. **(B)** Growth impact of 10 μM PD98059 for 24 h in PANC-1 cells, expressed in percentage of control as mean ± SD of three independent experiments. **p* < 0.05 by unpaired Student’s t-test.
