# Supplementary figures and images for: Integrating Gemcitabine-Based Therapy With AdipoRon Enhances Growth Inhibition in Human PDAC Cell Lines
Source: Front Pharmacol. 2022 Feb 22;13:837503. doi: 10.3389/fphar.2022.837503 (PMC8902254; doi:10.3389/fphar.2022.837503)

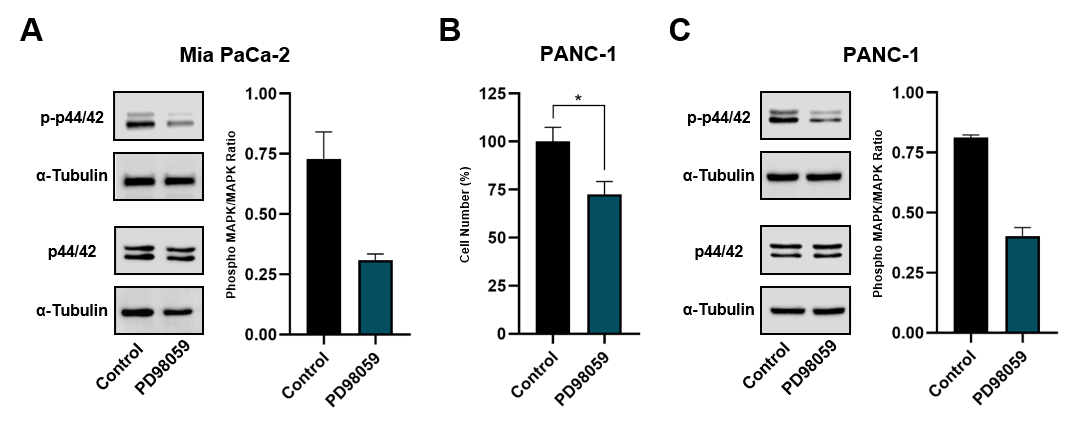

Supplement: Supplementary file 1 [file Image2.TIF]

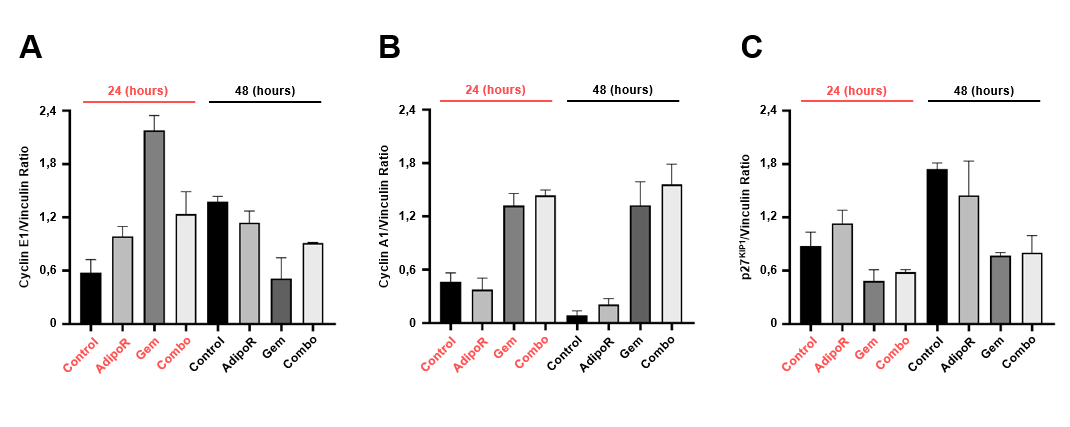

Supplement: Supplementary file 2 [file Image1.TIF]
